# Association between choriocapillaris flow deficit and choroidal neovascularization activity in eyes with myopic choroidal neovascularization

**DOI:** 10.1038/s41598-021-01557-z

**Published:** 2021-11-09

**Authors:** Sato Uematsu, Hirokazu Sakaguchi, Kaori Sayanagi, Yasushi Ikuno, Ayako Yokoyama, Tomoko Asai, Yoko Fukushima, Chikako Hara, Susumu Sakimoto, Kohji Nishida

**Affiliations:** 1 Department of Ophthalmology, Osaka University Graduate School of Medicine, Suita, Japan; 2 Division of Ophthalmology, Itami City Hospital, Itami, Japan; 3 Department of Advanced Device Medicine, Osaka University Graduate School of Medicine, 2-2 Yamadaoka, E-7, Suita, Osaka 565-0871 Japan; 4 Ikuno Eye Center, Osaka, Japan; 5 Eye Center, Sakai City Medical Center, Sakai, Japan; 6 Asai Eye Clinic, Amagasaki, Japan; 7 Integrated Frontier Research for Medical Science Division, Institute for Open and Transdisciplinary Research Initiatives (OTRI), Osaka University, Suita, Japan

**Keywords:** Retinal diseases, Eye diseases

## Abstract

Although choriocapillaris flow deficit (CFD) around choroidal neovascularization (CNV) is less associated with CNV activity in myopic eyes, no reports are investigating its size as an indicator of CNV activity. We investigated the relationship between CFD and high myopia-related CNV. In this retrospective, observational study, patients underwent optical coherence tomography angiography (OCTA) with split-spectrum amplitude-decorrelation angiography for diagnosing pathological myopic CNV (mCNV); CFD features around CNV margins were evaluated. Of the 33 eyes (30 patients), 11 (33.3%) had active mCNV, and 22 (66.7%) had inactive CNV. Six eyes (18.2%) were treatment-naïve, while the remainder previously underwent anti-vascular endothelial growth factor therapy. On OCTA, blood flow signals were detected in CNV in the outer retinal layer in 28 (84.8%) eyes, including all active cases (11 cases) and 17 (77.3%) of 22 inactive cases. CNV flow signal size correlated significantly with activity (P < 0.001). CFD around CNV was observed in 24 eyes (72.7%), including all active cases (11 cases) and 13 (59.1%) of 22 inactive cases. CFD size correlated significantly with CNV activity (P < 0.001). The size of both the CFD area around CNV and CNV flow signal area are useful indicators of CNV activity in eyes with mCNV, which may help determine treatment timing.

## Introduction

Pathologic myopia, defined as high myopia exceeding − 6.0 diopters (D) or an axial length of ≥ 26 mm with complications at the posterior segment, such as chorioretinal atrophy and posterior staphyloma^1^, is a common cause of low vision and visual impairment^2^, particularly in East Asia^3–5^. Choroidal neovascularization (CNV) is a major cause of visual loss in patients with pathologic myopia^6,7^. The natural course of long-term visual outcomes in patients with CNV owing to pathologic myopia (mCNV) is unfavorable. The visual acuity (VA) of most patients with mCNV decreases to 20/200 or less within 5–10 years after the onset of CNV secondary to the development of CNV-related macular atrophy^8^, which is associated with defects in the Bruch’s membrane^9^.

The first-line treatment for mCNV includes intravitreal anti-vascular endothelial growth factor (VEGF) injections. Recent clinical trials have reported safe, substantial VA gains with the use of anti-VEGF agents in patients with mCNV^10,11^. However, mCNV often relapses repeatedly, and a mean of 2.0‒4.0 anti-VEGF (ranibizumab) treatments (Lucentis, Genentech, San Francisco, CA, USA) was required in the RADIANCE study^10^, and 4.2 treatment with aflibercept (Eylea, Regeneron Pharmaceuticals, Tarrytown, NY, USA) were required in the first year in the MYRROR study^11^.

Non-invasively determining the proper timing of retreatment is therefore important to avoid overdoses of anti-VEGF drugs. Fluorescein angiography (FA) is the gold standard for diagnosing mCNVs^12^. FA is essential for obtaining information about the presence, type, area, and activity of mCNV and helps to exclude other disorders. It is generally used to identify the fovea, assess retinal thickness and the presence of extracellular fluid, and establish a baseline for judging future treatment responses^13^. However, FA might be unsuitable for repeat examinations because of its invasiveness. Optical coherence tomography (OCT), which is non-invasive, plays a major role in the follow-up of patients with CNV. However, it is sometimes difficult to determine CNV recurrence based on OCT alone.

OCT angiography (OCTA) is a new tool for the non-invasive detection of vascular flow. It has been reported that OCTA is useful for identifying CNV^14,15^, monitoring CNV during follow-ups after anti-VEGF treatment^16^, and analyzing vascular morphology^17^ in age-related macular degeneration (AMD) and mCNV^18^. Recently, several studies have investigated the usefulness of OCTA as a predictor of CNV activity. Coscas et al.^19^ examined the usefulness of four OCTA findings as predictors of CNV activity in AMD cases and reported that small branching vessels are effective indicators of CNV activity, while a hypointense halo around CNV is a less effective indicator. Additionally, Li et al.^20^, who conducted the same study in mCNV cases, argued that small branching vessels were a major predictor, CNV signal pattern and loop/anastomoses were minor predictors, and a choroid dark halo around the CNV was a poor predictor of CNV activity.

Another recent discovery using OCTA was the identification of choriocapillaris flow deficit (CFD). Several investigators have described CFD in eyes with nonexudative AMD, geographic atrophy, and high myopia^21–24^. Although previous studies have reported that the presence/absence of CFD around CNV has been less associated with CNV activity in the eyes with mCNV^20,24^, there have been no reports investigating the size of the CFD around CNV as an indicator of CNV activity.

Therefore, our study investigated the relationship between the size of the CFD and CNV activity in mCNV cases, with or without anti-VEGF treatment, using OCTA to facilitate the determination of treatment timing.

## Results

### Patient demographics

Among the 78 patients, 33 eyes of 30 patients (22 women, eight men; mean age, 60.5 ± 14.5 years; range, 20–80 years) matched the inclusion criteria. The mean spherical equivalent refractive error was − 13.0 ± 4.2 D (range, − 22.0 to 7.5 D) after the exclusion of 20 pseudophakic eyes. The mean logarithm of the minimum angle of resolution (logMAR) best-corrected decimal VA (BCVA) was 0.15 ± 0.24 (range, − 0.18 to + 0.70). The mean axial length was 29.5 ± 1.6 mm (range, 26.6‒33.3 mm). Eleven (33.3%) eyes had active mCNV, and 22 (66.7%) eyes had inactive CNV. Fourteen eyes had subfoveal CNV, and 19 eyes had juxtafoveal CNV. Ten (37.0%) eyes of 27 previously treated eyes had previous intravitreal bevacizumab injections, 10 (37.0%) had previous intravitreal ranibizumab (IVR) injections, and seven (25.9%) had previous intravitreal aflibercept (IVA) injections before the study period. Six (18.2%) of 33 eyes were treatment-naïve and received IVR treatment during the follow-up period. Since the last anti-VEGF treatment, the mean time elapsed was 22.8 ± 26.5 months (range, 0–101 months) for all examinations.

### OCTA of mCNV eyes

**Figure 1:**
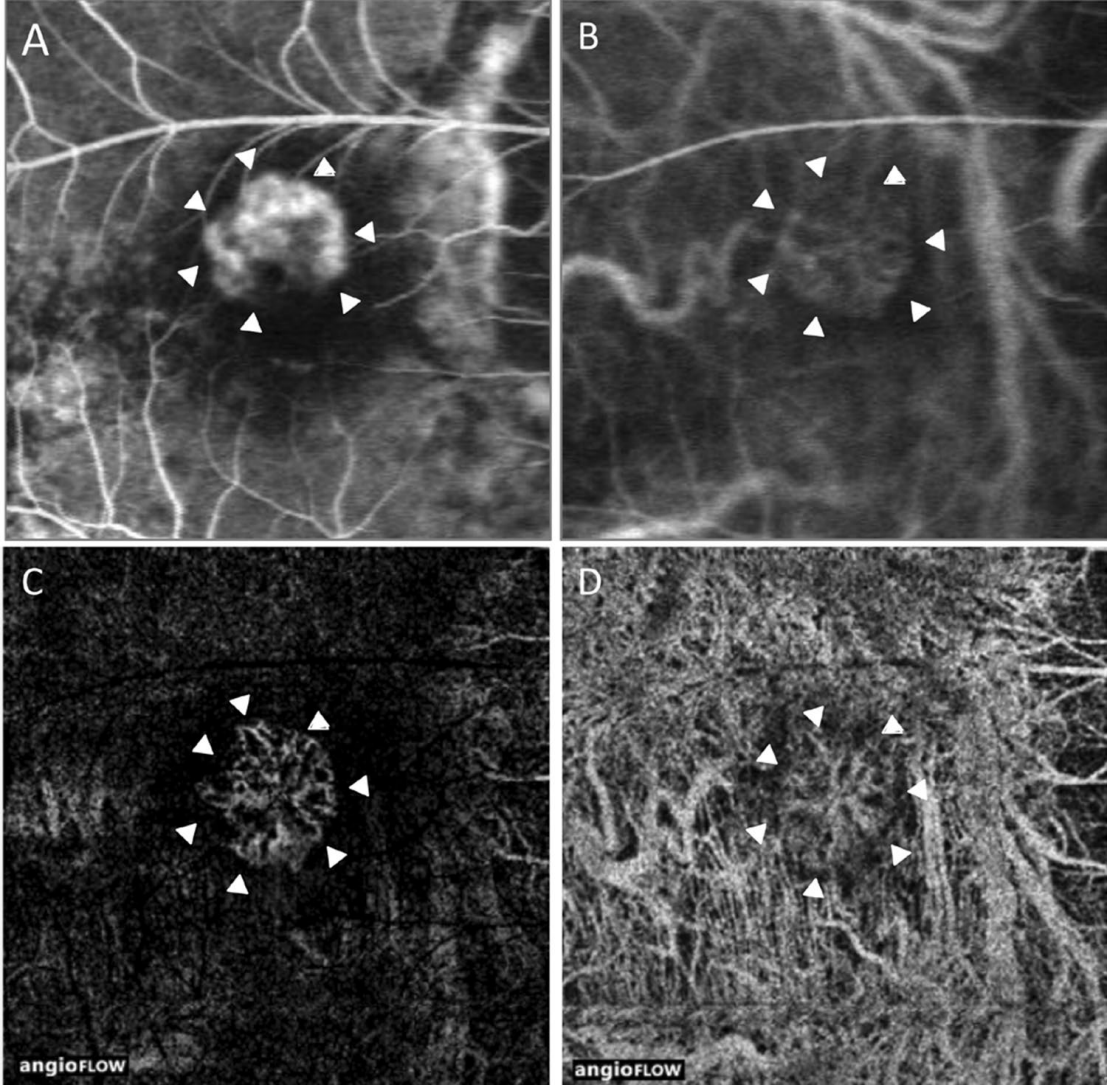
Fluorescein (FA) and indocyanine green angiography (IA) and optical coherence tomography angiography (OCTA) images of choroidal neovascularization (CNV) owing to pathologic myopia of the right eye in a 68-year-old man without anti-vascular endothelial growth factor treatment. Arrowheads (**A**‒**D**) indicate the location of CNV. (**A**) An early-phase FA image (34 s) shows hyperfluorescence corresponding to CNV. (**B**) An early-phase IA image (44 s) shows faint hyperfluorescence from CNV, which coincides with CNV in the FA image. (**C**) The outer retinal layer on the OCTA image of the identical retinal field on FA and IA images shows a flow signal. The location is similar to that observed with FA, and the vascular pattern is similar to that observed with IA. (**D**) An OCTA image of the choriocapillaris layer shows a vascular complex at the same location as in the outer retinal layer on an OCTA image. The choriocapillaris flow deficit around CNV is also detected as a dark area around CNV.

Twenty-eight (84.8%) eyes had flow signals on OCTA images corresponding to the location of CNV in the outer retinal layer (Fig. 1), and five (15.2%) eyes did not have a positive signal in the outer retinal layer on OCTA images. Of the 11 (100%) eyes of 11 active cases, six were treatment-naïve, and five had a recurrence, and 17 (77.3%) eyes of the 22 inactive cases had flow signals (*P* = 0.144). The mean size of the flow signal was 0.25 ± 0.27 mm^2^. Although none of the parameters, including age, logMAR BCVA, axial length, activity, location, and duration from the last anti-VEGF injection, were significantly associated with the presence or absence of flow signals in CNV (Table 1), the size of the flow signal area significantly correlated with CNV activity in univariate analyses (P < 0.001; Table 2).

**Table 1:** Comparison of characteristics between myopic choroidal neovascularization eyes with or without blood flow signals.

Clinical parameters	Positive	Negative	P-value
Total (%)	28 (84.8)	5 (15.2)	
Sex (female/male)	19/7	3/1	1.000^§^
Age (years)	60.5 ± 14.9	60.3 ± 13.5	0.971^†^
BCVA (LogMAR)	0.15 ± 0.25	0.13 ± 0.18	0.939^‡^
Axial length (mm)	29.3 ± 1.5	30.5 ± 2.2	0.149^†^
Activity (active/inactive)	11/17	0/5	0.144^§^
Location (subfoveal/juxtafoveal)	14/14	0/5	0.057^§^
Follow-up duration from the last injection of anti-VEGF (months)	23.0 ± 27.8	21.6 ± 19.4	0.687^‡^

*OCTA* optical coherence tomographic angiography, *BCVA* best-corrected visual acuity, *VEGF* vascular endothelial growth factor, *LogMAR* logarithm of the minimum angle of resolution. ^†^Student’s *t*-test; ^‡^Wilcoxon rank sum test; ^§^Fisher's exact test.

**Table 2:** Univariate regression analyses of the median size of flow signal areas and other factors in optical coherence angiography of myopic choroidal neovascularization.

Clinical parameters	Regression coefficient (SE)	P-value	Standardized estimate
Age (years)	0.004 (0.004)	0.358	0.165
BCVA (LogMAR)	0.391 (0.213)	0.076	0.313
Axial length (mm)	− 0.026 (0.036)	0.464	− 0.136
Activity (active/inactive)	− 0.350 (0.094)	< 0.001	− 0.555
Location (subfoveal/juxtafoveal)	0.168 (0.104)	0.117	0.278
Follow-up duration from the last injection of anti-VEGF (months)	− 0.003 (0.002)	0.111	− 0.282

*SE* standard error, *OCTA* optical coherence tomographic angiography, *BCVA* best-corrected visual acuity, *VEGF* vascular endothelial growth factor, *LogMAR* logarithm of the minimum angle of resolution.

### CFD around CNV

**Figure 2:**
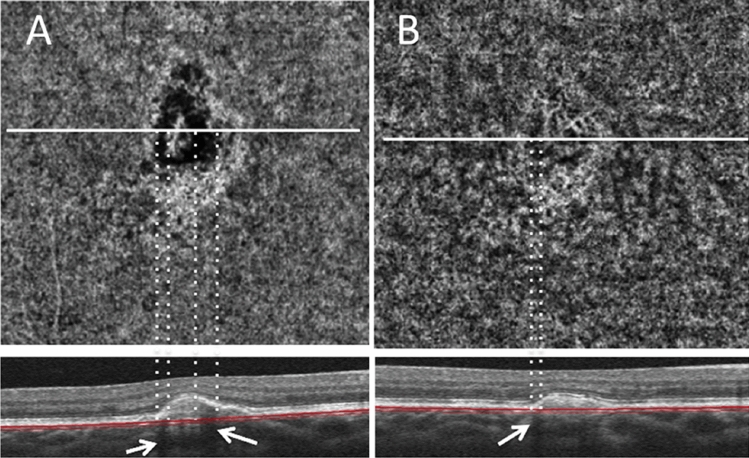
Optical coherence tomography angiography images of the choriocapillaris layer with horizontal cross-sections from a 36-year-old man with myopic choroidal neovascularization (CNV). (**A**) The patient had previously been treated with intravitreal ranibizumab (IVR) injection. Eight months after the initial IVR injection, the CNV complex was re-enlarged, and a choriocapillaris flow deficit (CFD) was observed around CNV. The CFD nearly corresponds to the dark area on a B-scan OCT image (arrows). A second injection of IVR was administered. (**B**) One year after the second IVR injection, the CNV complex and CFD remained, but the CFD decreased in size.

**Figure 3:**
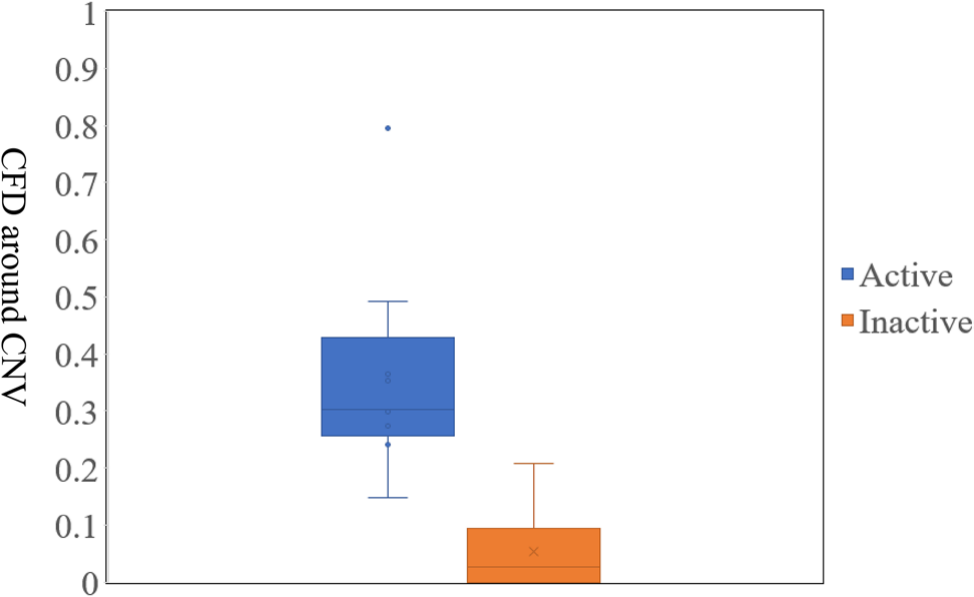
The box-and-whisker diagram of the activity (active/inactive) and size of the choriocapillaris flow deficit.

CFD around CNV was observed in 24eyes (72.7%). All eyes with 11 eyes of active CNVs had CFD around CNV. The mean size of the CFD was 0.18 ± 0.18 mm^2^. Figure 2 shows a representative image of CFD around CNV. Based on the univariate analysis, none of the parameters other than activity, such as age, logMAR BCVA, axial length, location, and time since last anti-VEGF injection, were significantly associated with the presence of CFD (Table 3); however, the size of the CFD significantly correlated with CNV activity (P < 0.001; Table 4, Fig. 3).

**Table 3:** Comparison of characteristics between eyes with or without choriocapillaris flow deficit around choroidal neovascularization.

Clinical parameters	Positive	Negative	P-value
Total (%)	24 (72.7)	9 (27.3)	
Gender (female/male)	17/5	5/3	0.643^§^
Age (years)	59.7 ± 15.1	62.6 ± 13.2	0.636^†^
BCVA (LogMAR)	0.18 ± 0.27	0.07 ± 0.15	0.148*
Axial length (mm)	29.2 ± 1.4	30.3 ± 1.9	0.086^†^
Activity (active/inactive)	11/13	0/9	0.015^§^
Location (subfoveal/juxtafoveal)	12/12	2/7	0.241^§^
Follow-up duration from the last injection of anti-VEGF (months)	18.1 ± 23.6	35.6 ± 30.8	0.052^‡^

*OCTA* optical coherence tomographic angiography, *BCVA* best-corrected visual acuity, *VEGF* vascular endothelial growth factor, *LogMAR* logarithm of the minimum angle of resolution. ^†^Student’s *t*-test; *Welch’s *t*-test; ^‡^Wilcoxon rank sum test; ^§^Fisher's exact test.

**Table 4:** Univariate linear regression analyses of the median size of the choriocapillaris flow deficit and other factors in optical coherence angiography of myopic choroidal neovascularization.

Clinical parameters	Regression coefficient (SE)	P-value	Standardized estimate
Age (years)	0.001 (0.002)	0.628	0.008
BCVA (LogMAR)	0.156 (0.132)	0.247	0.208
Axial length (mm)	− 0.031 (0.021)	0.142	− 0.270
Activity (active/inactive)	− 0.212 (0.057)	< 0.001	− 0.559
Location (subfoveal/juxtafoveal)	0.122 (0.061)	0.055	0.337
Follow-up duration from the last injection of anti-VEGF (months)	− 0.002 (0.001)	0.053	− 0.339

*SE* standard error, *OCTA* optical coherence tomographic angiography, *BCVA* best-corrected visual acuity, *VEGF* vascular endothelial growth factor, *LogMAR* logarithm of the minimum angle of resolution.

## Discussion

Our study investigated the relationship between the size of CFD and CNV activity in eyes with pathological myopia. We found that 77.3% of the eyes with inactive mCNV still had vessel flow signals in the outer retinal and choriocapillaris layers, which matched the position of the CNV on previously obtained FA images, indicating that the vessel flow in CNV had been present for years. Interestingly, the magnitude of the blood flow signal in the active CNV did not differ from that in the inactive CNV, indicating that most inactive CNVs without subretinal fluid or edema still showed vessel flow on OCTA images; however, other investigations showed that the flow area size had decreased in inactive CNV cases. Thus, the residual blood flow signal itself was not a reason for using anti-VEGF drugs, although OCTA could help diagnose mCNV. However, persistent blood flow may have played a role in the recurrence. Further studies are needed to clarify whether the presence of flow signals affects the recurrence of mCNV.

Regarding the relationship between OCTA and FA for the eyes with mCNV, using OCTA images, we detected positive signals in 100% (11 eyes) of active mCNV cases in the deep retinal layer, which was similar to the results of Miyata et al.^25^, who reported that 94.1% of the mCNV cases could be observed using OCTA alone and that the size of the CNV on FA and OCTA significantly correlated. These investigators defined mCNV as a split-spectrum amplitude-decorrelation angiography (SSADA)-positive area in the outer retinal layer, which agreed with our definition. The outer retinal layer normally has a darker background than the choriocapillaris layer, making it easier to detect.

Several reports have discussed finding a dark rim on indocyanine green angiography (IA) test results. Scheider et al.^26^ first documented a dark rim, defined as a circular background of hypofluorescence surrounding the neovascular membrane in early phase IA images. Other investigators histopathologically showed that the dark rim was owing to blockage of the IA light by an intense accumulation of the retinal pigment epithelium (RPE) around experimentally induced CNV in monkey eyes^27^. Kang et al.^28^ reported that the absence of a dark rim at baseline was a risk factor for mCNV recurrence. In contrast, the appearance of CFD around CNV in OCTA images in our study differed from the dark rim previously described in IA images because the size of the dark rim should not change after treatment since its main body is an accumulation of RPEs, unlike CFD around CNV^27^. We believe that the dark rim and CFD around CNV represent different findings.

The most interesting finding of our study was the significant correlation between the size of the CFD around CNV and CNV activity. Some studies have reported that the presence or absence of CFD around CNV is not relevant to CNV activity^19,20^. Alagorie et al.^29^ reported that the CFD around CNV was significantly greater in eyes with AMD than in healthy controls; moreover, there was no significant correlation between CFD around CNV and age, VA, or CNV area. However, to the best of our knowledge, no previous report has examined the association of the size of the CFD around CNV and CNV activity to date. Although further investigation with a larger number of eyes is necessary to verify our findings, our results indicate that, at least for mCNV, CFD around CNV could be an indicator of CNV activity.

The origin of CFD around CNV remains uncertain; however, we hypothesize that the RPE or thickened retinal tissue blocks the light and reduces the signal from the choriocapillaris, or there is no or very slow blood flow surrounding CNV. The first hypothesis seems reasonable because of the presence of shadows around the CNV, as shown in the horizontal cross-sections (Fig. 2, arrows). If the blood vessels completely disappear, they usually do not recover with time. However, if the signal of blood flow that should be there is shaded by CNVs and is below the detection limit of OCTA, it is possible that the OCTA can detect blood flow and restore the signal. Iida et al. used the same principle to explain the dark rim of idiopathic CNVs^30^. Nevertheless, the origin of the CFD remains unknown. Some dark areas seemed to coincide with the shadow of the B-scan on conventional OCT images. However, shadows do not always appear. For instance, Xie et al.^31^ recently reported that a dilated choroidal vein beneath CNV was observed in 30% of mCNV eyes. One mechanism underlying the CFD around CNV might be choriocapillaris exclusion by the choroidal vein. Further studies are required for verifying these findings.

This study has some limitations. The number of eyes used to characterize the disease course was small, both with and without treatment. Additionally, no highly myopic patients without CNV were available for comparison. As this was a retrospective observational study, further evidence is needed to show more definitively that the remaining blood flow on OCTA images was responsible for recurrence. An analysis of only post-treatment cases may allow for a more rigorous evaluation of the effects of anti-VEGF therapy on CFD and flow signals. Currently, OCT-B scans and/or FA are better when used simultaneously, although OCTA supports these conventional tools. Therefore, further studies are needed for investigating the morphological changes in CNV after anti-VEGF therapy.

In conclusion, our study documented the characteristics of mCNV and morphological changes with or without treatment based on OCTA images. Our findings show that OCTA images could be substituted for early-phase FA and IA images, although the OCTA images do not always correspond precisely with CNV activity. Nonetheless, OCTA images can noninvasively provide results useful for identifying CNV recurrence and may facilitate the determination of the timing of mCNV treatment in the clinic.

## Methods

Our study was performed according to the ethical standards of the Declaration of Helsinki. The institutional review board of Osaka University Hospital approved the study protocol. Informed consent was obtained in the form of an opt-out option on the website.

We retrospectively studied 78 eyes of 46 consecutive patients with high myopia who underwent OCTA between September 2014 and March 2015 at Osaka University Hospital. The inclusion criteria were the presence of high myopia (defined as − 6.0 D or an axial length of ≥ 26 mm) and a history or presence of mCNV. The exclusion criteria were the presence of punctate inner choroidopathy, CNV owing to diseases other than myopia, poor fixation, CNV outside a 3 × 3 mm^2^ square on an SSADA image, and CNV too large to fit within the 3 × 3 mm^2^ area.

Retina specialists (Y.I. and S.U. with 25 years and 10 years of professional experience, respectively) diagnosed mCNV based on the findings of fundus photographs, conventional OCT images, and FA images. BCVA was measured and converted to logMAR for analysis. The Zeiss IOL Master (Carl Zeiss Meditec, Oberkochen, Germany) was used for measuring the axial length.

### Assessing mCNV

The study included patients with active and inactive mCNVs. Inactive CNV was defined as CNV that had been detected or treated previously, with no active signs on examination in the present study. Active signs were defined as dye leakage on FA images using the Heidelberg Retina Angiograph 2 or the presence of subretinal fluid or intraretinal cysts on OCT images (Cirrus HD-OCT, Carl Zeiss Meditec, Dublin, CA, USA) or a swept-source OCT system (Topcon, Tokyo, Japan). Early-phase FA images (within 2 min after the start of imaging) were used to locate mCNV on OCTA images.

### CNV flow signal and CFD measurements

RTVue XR Avanti (Optovue, Fremont, CA, USA) with the AngioVue mode was used to obtain 3 × 3 mm^2^ and 6 × 6 mm^2^ OCTA images. Four layers of en-face vessel images were generated (e.g., superficial retinal layer, deep retinal layer, outer retinal layer, and choroidal capillary layer) using the AngioVue mode, as previously described by the SSADA algorithm^32^. Three examiners (S.U., K.S., and A.Y.) measured the flow signal area of CNV (area A) using ImageJ software (National Institute of Health, Bethesda, MD, USA) in the choriocapillaris layers of OCTA images. Area B included both CFD, defined as a dark hypo-signal area around CNV, and CNV, which was measured similarly. The size of the CFD around CNV was calculated by subtracting area A from area B. The number of pixels was used to calculate the area. The presence or absence of the CNV flow signal was assessed by whether the signal was detected in OCTA images. A CFD area was evaluated as "presence of CFD" if it accounted for 20% or more of the total area (area B). When the OCTA images of multiple visits were available, images from the first visit were used for all analyses. The average values from the three examiners were used in this study.

### Anti-VEGF treatment

The study included both naïve patients and patients that had been treated with anti-VEGF agent injections. Retina specialists administered the following intravitreal injections of anti-VEGF treatment to patients with mCNV: 1.0 mg of bevacizumab (Avastin, Genentech, San Francisco, CA, USA), ranibizumab (0.5 mg), or aflibercept (2.0 mg) according to a pro re nata regimen when mCNV was diagnosed as active. Bevacizumab was used between May 2006 and August 2013 before the Japanese government approved ranibizumab or aflibercept for mCNV treatment; ranibizumab was used after August 2013, and aflibercept was used after September 2014. The use of bevacizumab was approved by the institutional review board of Osaka University Hospital.

### Statistical analyses

Statistical analyses were performed using the JMP statistical software (SAS Institute, Cary, NC, USA). Fisher’s exact test was used for analyzing the non-parametric data. Student’s *t*-test and one-way analysis of variance were used for parametric numerical data, and the Wilcoxon *t*-test and Kruskal–Wallis test were used for nonparametric numerical data. A *P*-value of < 0.05 was considered significant. The multivariate linear regression analysis was used when multiple variables were found to be statistically significant.

## Data Availability

All data generated or analyzed during this study are included in this published article.
